# Hydroxyethyl Cellulose-Based Hydrogels as Controlled Release Carriers for Amorphous Solid Dispersion of Bioactive Components of *Radix Paeonia Alba*

**DOI:** 10.3390/molecules28217320

**Published:** 2023-10-28

**Authors:** Abid Naeem, Chengqun Yu, Xiaoli Wang, Mingyan Peng, Yi Liu, Yali Liu

**Affiliations:** 1 Key Laboratory of Modern Preparation of Traditional Chinese Medicines, Ministry of Education, Jiangxi University of Chinese Medicine, Nanchang 330004, China; yuchengqun@jxutcm.edu.cn (C.Y.); 202001012021@jxutcm.edu.cn (M.P.);; 2 Key Laboratory of Pharmacodynamics and Quality Evaluation on Anti-Inflammatory Chinese Herbs, Jiangxi Administration of Traditional Chinese Medicine, Nanchang Medical College, Nanchang 330006, China; 3 Hubei Key Laboratory of Embryonic Stem Cell Research, Taihe Hospital, Hubei University of Medicine, Shiyan 442000, China; 4 Key Laboratory of Pharmacodynamics and Safety Evaluation, Health Commission of Jiangxi Province, Nanchang 330006, China

**Keywords:** hydrogels, phytoconstituents, UHPLC-Q-TOF-MS, solid dispersion, traditional Chinese medicine, antimicrobial agents

## Abstract

*Radix Paeoniae Alba* (RPA) has been used extensively in Chinese traditional medicine to treat gastrointestinal disorders, immune-modulating diseases, cancers, and numerous other conditions. A few of its active components include paeoniflorin, albiflorin, lactiflorin, and catechin. However, their therapeutic effectiveness is compromised by poor pharmacokinetic profiles, low oral bioavailability, short half-lives, and poor aqueous solubility. In this study, hydroxyethyl cellulose-grafted-2-acrylamido-2-methylpropane sulfonic acid (HEC-*g*-AMPS) hydrogels were successfully prepared for the controlled release of *Radix Paeonia Alba*-solid dispersion (RPA-SD). A total of 43 compounds were identified in RPA-SD using UHPLC-Q-TOF-MS analysis. The hydrogel network formation was confirmed by FTIR, TGA, DSC, XRD, and SEM. Hydrogels’ swelling and drug release were slightly higher at pH 1.2 (43.31% swelling, 81.70% drug release) than at pH 7.4 (27.73% swelling, 72.46% drug release) after 48 h. The gel fraction, drug release time and mechanical strength of the hydrogels increased with increased polymer and monomer concentration. Furthermore, the hydrogels were porous (84.15% porosity) and biodegradable (8.9% weight loss per week). Moreover, the synthesized hydrogels exhibited excellent antimicrobial and antioxidative properties.

## 1. Introduction

Herbal remedies have traditionally been used to treat a wide range of ailments throughout history. A limited number of dosage forms are available for herbal medicines due to their complex composition [1]. The majority of herbal medicines are prepared in the form of crude extracts [2]. They can be dried or stored fresh and contain both primary and secondary metabolites. Several primary metabolites are present in plants, including amino acids, proteins, lipids and sugars [3]. The secondary metabolites produced by plants are both bioactive and non-bioactive compounds [4]. Herbal extracts can lose many of their pharmacological properties after many of their active components have been separated and purified [5]. The therapeutic efficacy of herbal extracts is reduced after extraction due to decreased pharmacokinetic interactions between the active ingredients [6,7]. Bioactive substances in their pure form have a lower pharmacokinetic profile than herbal extracts of the same composition. The pharmacological properties of herbal medicines are often influenced by synergistic interactions among components with different properties and functions [8].

Small molecules found in herbal extracts may facilitate the solubilization of coexisting bioactive substances [9]. There may be an increase in the water solubility of herbal extracts; however, this does not necessarily imply that each constituent of the extract is dissolved in water. The compounds seem soluble rather than more solubilized [10]. In most cases, herbal extracts are prepared as powders and then reconstituted in water before consumption [11,12]. Herbal extracts contain a variety of hydrophilic secondary metabolites, which makes powdered extracts an amorphous solid dispersion containing a high amount of bioactive compounds [13,14].

*Radix Paeoniae Alba* (RPA) is a well-known ranunculus family herb in China, Korea, and Japan that has been widely used for more than 1200 years for treating several diseases, including gastrointestinal disorders, cardiovascular diseases, liver protection, antioxidants, immunomodulators, cancer, etc. [15]. It is often used with conventional medical treatment and is compatible with traditional Chinese medicine. Many prescription medicines, such as Huangqi Jianzhong Tang and Xiaoyaosan, contain *Radix Paeoniae Alba* plant extracts as adjuvants [16]. There are several major chemical components in *Radix Paeoniae Alba*, including monoterpenes, amino acids, polysaccharides, volatile oils, tannins, flavonoids, and organic acids [17]. These agents are limited in their therapeutic efficacy due to the fact that they have poor pharmacokinetic profiles (low oral bioavailability, rapid systemic clearance, short half-lives), as well as poor physical properties (including poor water solubility and decreased stability). Some of these limitations can be overcome by developing a controlled-release dosage form, however, no suitable controlled or sustained-release dosage forms are available for *Radix Paeoniae Alba* to maximize its therapeutic efficacy and reduce its side effects.

Over the past few decades, scientists around the world have developed advanced delivery systems that have improved the clinical safety, effectiveness, and ease of administration of traditional medicinal agents [18,19]. It has been demonstrated that controlled delivery systems are more efficient than traditional dosage forms, mainly due to the reduction of side effects and dosage. Furthermore, they also improve treatment compliance. Hydrogels are three-dimensional (3D), water-swollen and crosslinked hydrophilic networks having resistance to dissolving in water and biological media [20]. Due to their high water content, hydrogels mimic the natural environment found in tissues [21]. Hydrophilic groups in polymer chains’ backbone, including amino, hydroxyl, and carboxyl groups, trigger water absorption. Biodegradable and hydrophilic gels are chemically constructed using natural and synthetic polymers [22]. Hydrogels have the advantage of being flexible and smooth, which decreases inflammation in the tissues surrounding them. Porosity is determined by many factors, including its attraction to aqueous environments where it has swollen. The density of crosslinking agents within the gel matrix is also important. The porous hydrogel structure facilitates the efficient loading of bioactive substances into the matrix, allowing for controlled drug release based on the diffusion coefficients of small and large molecules [23].

Cellulose is the most abundant polysaccharide in nature and possesses several desirable properties, including biodegradability, biocompatibility, non-toxicity, low cost, and stability. It is a linear polysaccharide that consists of 1,4-β-d-glucopyranosyl units [24]. Molecular chains of cellulose molecules form tightly bound networks that are stabilized by inter- and intrachain hydrogen bonds. The cellulose structure prevents it from being dissolved in water, limiting its profit potential. Hydroxyethyl cellulose (HEC) is a commercially important derivative of cellulose that is soluble in water. This material’s high biocompatibility, low toxicity, and non-immunogenicity make it suitable for various industrial, biophysical, and biotechnological applications, such as coatings, thickeners, pharmaceuticals stabilizers, and cosmetics [25]. 2-acrylamido-2-methylpropane sulfonic acid (AMPS) consists of sulfonic acids, amides, and carbonyls and forms hydrogels [26]. The hydrogel was crosslinked using ethylene glycol dimethacrylic acid (EGDMA) [27].

Hydrogels offer potential advantages for controlled drug delivery, so we loaded *Radix Paeonia Alba*-SD into these gels and investigated their physical-chemical properties. Swelling patterns and drug release mechanisms were investigated using different pH-valued solutions of phosphate buffer (pH 1.2 and pH 7.4). Hydrogel characteristics were examined using various techniques, including thermal properties, compatibility between ingredients, sol-gel, morphology and porosity, biodegradation rate, and mechanical characteristics. Furthermore, we evaluated the effect of polymer and crosslinker concentrations on hydrogels’ swelling and release behavior. *Radix Paeonia Alba*-solid dispersion-loaded hydrogels were also evaluated for their antioxidant and antimicrobial activities.

## 2. Results and Discussion

**Figure 1:**
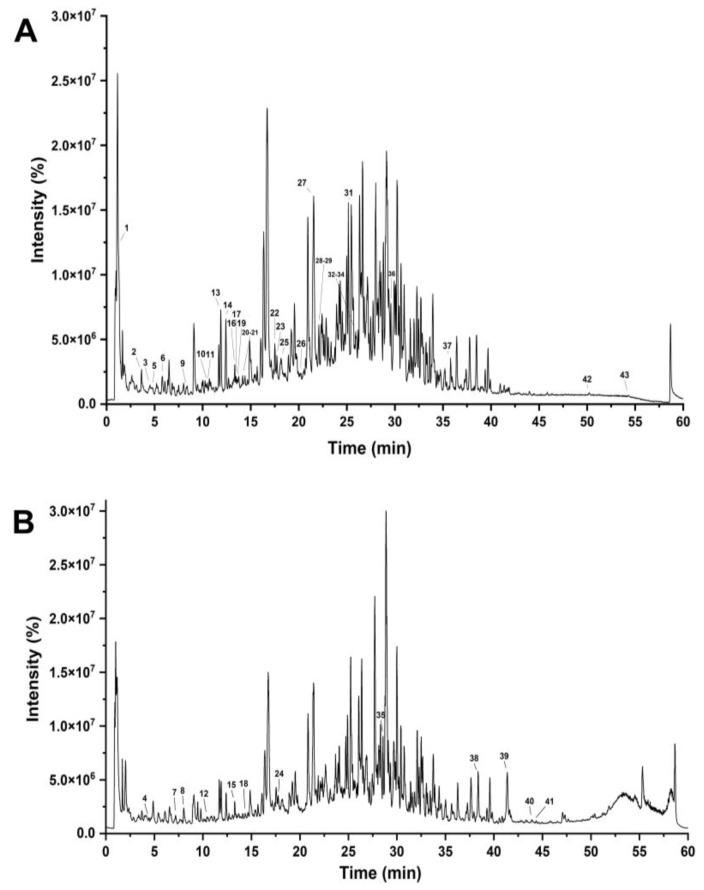
UHPLC–Q–TOF–MS chromatograms of RPA–SD in negative (**A**) and positive electrospray ionization modes (**B**).

RPA-SD mostly consists of isoflavones that are unique to the paeoniflorin family. It has been shown that these components have many biological effects, such as lowering blood pressure, preventing free radical damage, lowering blood sugar levels, reducing inflammation, and protecting nerve cells. The medicinal effects of *Radix Paeonia Alba* are thought to originate mostly from these constituents [28]. The UHPLC-Q-TOF-MS chromatograms are shown in Figure 1, and the identified compounds are listed in Table 1.

### 2.1. FTIR Spectral Analysis

**Figure 2:**
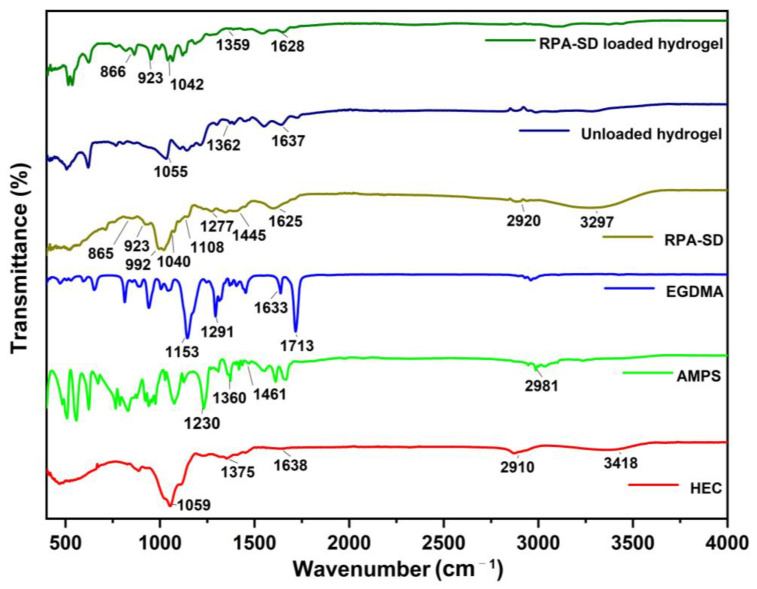
FTIR spectra of HEC, AMPS, EGDMA, RPA-SD, unloaded and RPA-SD loaded hydrogels.

FTIR spectra of HEC, AMPS, EGDMA, RPA-SD, unloaded, and RPA-SD loaded hydrogels are depicted in Figure 2. According to the FTIR spectrum of AMPS, a vibration band was observed at 1461 cm^−1^, which was associated with CH_2_’s binding vibration. The bands observed at 1360 cm^−1^ corresponded to -C-O stretching vibrations, whereas the characteristic bands at 2981 cm^−1^ reflect -CH stretching of -CH_2_. The band observed at 1230 cm^−1^ is associated with the symmetric stretching of the S=O functional group [29]. The FTIR spectrum of EGDMA showed a major band at 1713 cm^−1^, which is due to the C=O stretching of acrylate. Furthermore, the bands at 1633, 1291, and 1153 cm^−1^ corresponded to stretching vibrations of C=C and C–O groups, respectively. These characteristics are mostly observed in both symmetrical and asymmetric ester chains [30]. Several stretching vibration absorption bands were observed in the HEC spectrum, such as the band near 1375 cm^−1^ belongs to C-OH, the band at 2910 cm^−1^ is associated with C-H stretching, the band at 3418 cm^−1^ belongs to -OH stretching vibration and the band at 1059 cm^−1^ belongs to C-O-C stretching vibrations. Other researchers have also reported similar results [31]. The RPA-SD spectrum shows the bands at 1108, 1040, and 992 cm^−1^, which are attributed to the stretching vibration of glycoside C-O. Furthermore, the band at 923 cm^−1^ is attributed to the stretching vibration of the glycoside ring and the band at 1277 cm^−1^ is attributed to the stretching vibration of ester group C-O-C [32]. In contrast to their parent components, hydrogels in their unloaded state displayed a different spectrum. The band at 1055 cm^−1^ was associated with the presence of a C-O-C group, while the band at 1067 cm^−1^ was associated with HEC. In addition, the vibration bands of the C-O group were observed at 1362 cm^−1^, which are associated with the AMPS. These new bands and functional groups indicate the presence of polymers and monomers in the cross-linked hydrogel network. FTIR analysis of the RPA-SD loaded hydrogel was performed to confirm the presence of the drug (RPA-SD) inside the hydrogel network. According to the results, there was a tensile vibration of RPA-SD’s glycoside ring at 923 cm^−1^ and the glycoside C-O at 1043 cm^−1^ in the hydrogel network. The presence of these bands confirmed the successful loading of the drug (RPA-SD) into the hydrogel network.

### 2.2. TGA Thermograms

**Figure 3:**
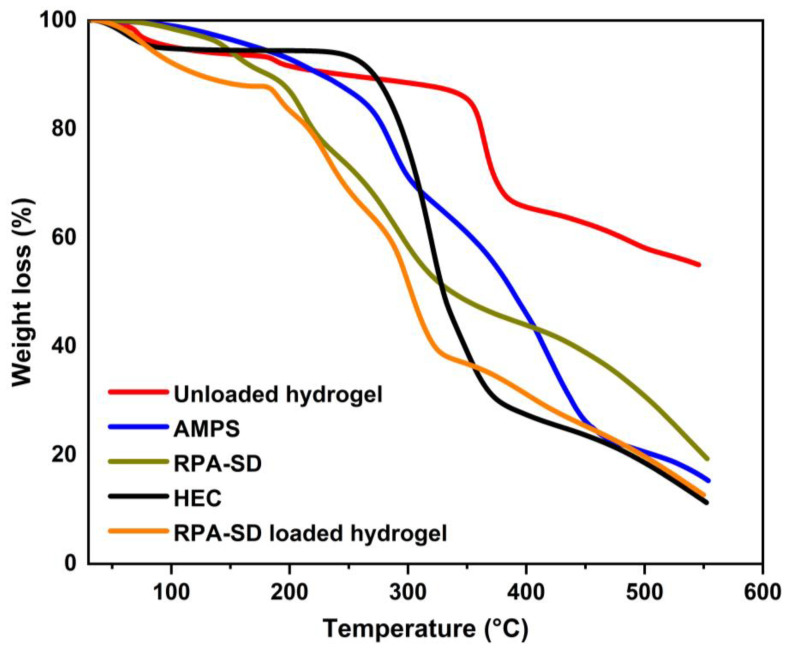
TGA thermograms of HEC, AMPS, RPA-SD, and synthesized hydrogels.

TGA was used to evaluate the thermal stability of the polymers and hydrogels (Figure 3). The TGA of AMPS revealed a weight loss of 6% as the temperature reached 208 °C; AMPS dehydrated to a further 20% between 210 and 250 °C, thus resulting in a weight loss of 20%; similarly, at 340 °C, the sulfonic acid group began to decompose, leading to a weight loss of 20% between 250 °C and 340 °C [33]. In the first stage of the TGA of HEC, the sample loses 4.1% of its weight due to the evaporation of the solvent in the sample. In the second stage, the sample loses 75.1% of its weight due to the decomposition of the sample. These outcomes are comparable to those observed by other groups [34]. The RPA-SD-loaded hydrogel was found to lose weight by 15% at 28 to 167 °C due to dehydration, followed by 44% at 167 to 314 °C as a result of polymer bond breakdown. Polymer networks disintegrated gradually at 319 °C and continued to degrade until the polymer skeleton was completely broken down. Unloaded hydrogels experienced a 14% weight loss at temperatures between 28 and 354 °C due to dehydration, followed by a 23% weight loss between 354 and 366 °C due to polymer bond breakdown. At a temperature of 366 °C, the polymeric network decomposes gradually and continues until the polymer’s backbone has been completely broken down. The thermal profile of hydrogels with a large residual weight suggests that the produced polymeric matrix is more resistant to thermal decomposition than the reactants over the full temperature range tested. In addition, because of the increased strength and contact between the polymer and monomer, a hydrogel will degrade at a higher temperature and at a slower rate than separate reactants [35]. The increased thermal stability results from the transfer of endothermic peaks to higher temperatures, forming a stiff network.

### 2.3. DSC Study

**Figure 4:**
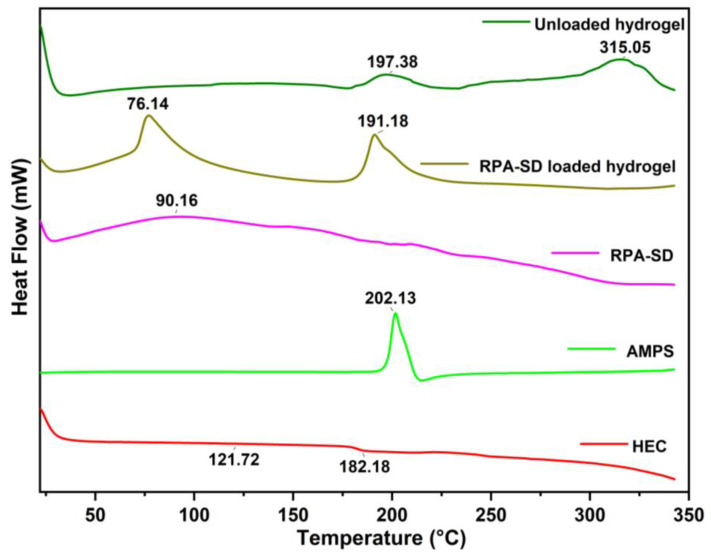
DSC thermogram of RPA-SD, AMPS, HEC and hydrogels.

The DSC thermogram of HEC exhibited a melting peak at 121.72 °C, corresponding to its glass transition temperature, and a degradation peak at 182.18 °C, corresponding to its decomposition temperature [36]. The AMPS results revealed a sharp endothermic peak at 202 °C, indicating dehydration [37]. An exothermic peak was observed at approximately 315 °C for the hydrogels obtained. These exothermic peaks of hydrogels suggest that the polymers were cross-linked, and the newly formed structure confers thermal stability on the formulation (Figure 4). A DSC analysis of the synthesized hydrogels indicates that the formulation has a higher glass transition temperature than its parent components. This demonstrates that the components are more compatible and form a rigid network structure due to increased hydrogen bonds between the molecules. As a result, the resulting polymeric network is more thermally stable [38].

### 2.4. XRD Studies

**Figure 5:**
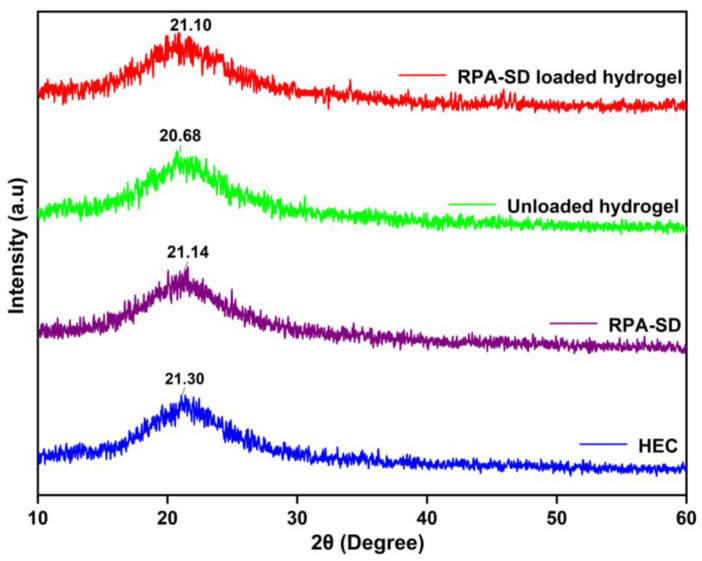
XRD of HEC, RPA-SD and developed hydrogels.

Figure 5 shows the XRD spectra of the polymer, RPA-SD, and synthesized hydrogels. The HEC diffractogram indicates that the polymer exhibits strong semi-crystalline properties and has a peak value of 2θ = 21.3°, which is consistent with findings by others [39]. The XRD diffractogram of unloaded hydrogel shows one broad peak at 2θ = 20.68°, which indicates an amorphous nature. On the other hand, the diffraction spectrum of pure RPA-SD displays peaks at 2θ = 21.14°, which indicates an amorphous nature. However, the diffractogram of RPA-SD loaded hydrogels shows only one broad peak at 2θ = 21.10°, whereas no other peaks of RPA-SD were displayed in its respective region, which might be due to the physical interaction of RPA-SD with the polymer matrix that affected drug purity and reduced crystal lattice characteristics.

### 2.5. Morphological Analysis

**Figure 6:**
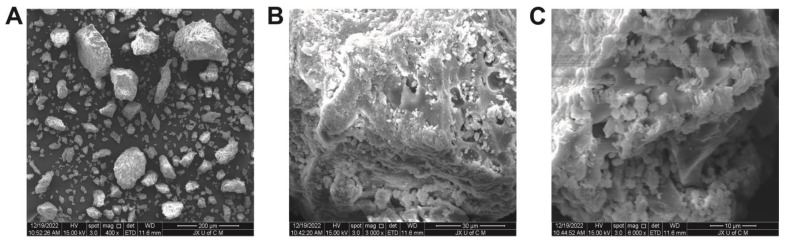
SEM micrographs of hydrogels at 400× (**A**), 3000× (**B**), and 6000× (**C**).

The surface morphology of the hydrogels, as illustrated in Figure 6, can be described as generally porous, rigid, and coarse in texture. The presence of such morphological characteristics indicates successful cross-linking of polymers [40]. These characteristics can be found in hydrogels intended for use in medicine. Micrographs of hydrogels show a rough, uneven, networked appearance containing micropores and macropores. Hydrogel networks have porous surfaces that allow fluids to diffuse throughout them. Furthermore, these pores facilitate the incorporation of drugs into the network and their transport to the target site. Micropores and macropores allow substantial amounts of aqueous fluid to be absorbed. Hydrogels contribute to the durability of polymer networks due to their solidity and smoothness.

### 2.6. Mechanical Properties Evaluation

Tensile strength (TS) and elongation at break (EAB) are the key mechanical properties of hydrogels and should be evaluated to ensure their success. The dynamic ionic crosslinking within the network-enabled HEC to self-recover rapidly and exhibit excellent fatigue resistance. HEC is a polymer with excellent mechanical properties, and other studies have found that tensile strength gradually increases with increased HEC content. The mechanical strength of the gel may decrease as the AMPS content increases, possibly due to an increase in electrostatic repulsion and osmotic pressure. The tensile strength of the material was also increased with an increase in EGDMA content (Table 2).

### 2.7. Sol-Gel Study

The non-crosslinked portion of hydrogels, known as the “sol fraction”, is distinguished from the crosslinked section, known as the “gel fraction”, during the polymerization procedure involving crosslinkers, monomers, and polymers [41]. Sols are formed during polymerization due to large amounts of one or more components and remain uncrosslinked since they lack reactive sites. It is, therefore, essential to determine the amount of crosslinking within the hydrogel and the amount of uncrosslinking within the hydrogel. We performed sol-gel testing on all of the prepared hydrogels with different compositions. The sol-gel analysis measures the uncrosslinked content of a hydrogel system. There is a wide variation in the gel fraction percentage under various material ratios, ranging from 80.17 to 97.12%. HEC exhibits good mechanical properties and adhesion to living surfaces [42]. When there are more AMPs, there is more space for chemical reactions to occur, which leads to gel formation. The crosslinking agent EGDMA is capable of causing the gel fraction to rise as the concentration of EGDMA increases.

### 2.8. Porosity Evaluation

**Figure 7:**
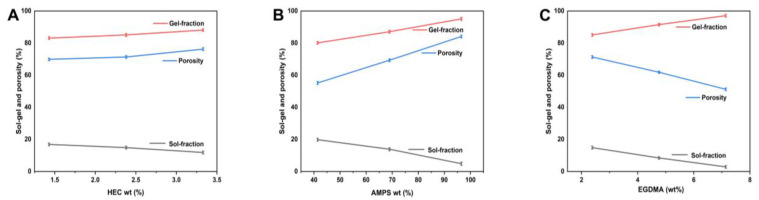
Impact of HEC (**A**), AMPS (**B**), and EGDMA (**C**) on the sol-gel fraction and porosity of hydrogels.

Figure 7 illustrates the porosity analysis of each synthesized hydrogel. Hydrogels’ porosity significantly impacts their ability to swell, load, and release drugs. Generally, larger pores result in more significant swelling, resulting in a greater volume of drug being loaded into and released from these pores. Since the reaction mixture is viscous, bubbles cannot escape, resulting in a greater porosity. Consequently, interconnected channels are formed, and porosity is increased. Porosity percentages varied between 51.22 and 84.15% under various reagent ratios. As a result, HEC has a good level of porosity. In general, the porosity of a substance increases as its HEC ratio increases. The results are consistent with other researchers [43]. Porosity decreases as EGDMA concentration increases due to the development of tight junctions and crosslinked bulk densities, affecting the drug’s release. AMPS concentration can be increased to increase porosity through the generation of stronger electrostatic forces through sulfonate groups. AMPS can form hydrophobic microregions due to their hydrophobic alkyl groups. It has been reported that the size of the pores and networks in hydrogels increases, which is in line with other reports [44].

### 2.9. Biodegradation of Synthesized Hydrogels

**Figure 8:**
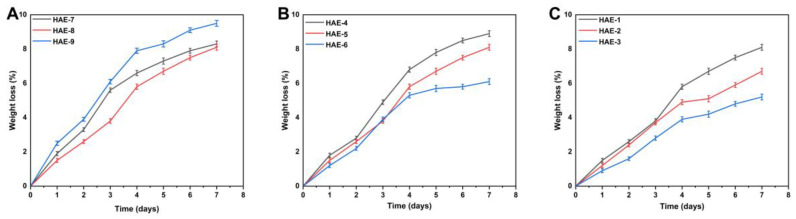
Influence of different composition ratios of ingredients on the in vitro biodegradation of hydrogels, such as HEC (HAE-7,8,9) (**A**), AMPS (HAE-4,5,6) (**B**), and EGDMA (HAE-1,2,3) (**C**).

Biodegradation studies are conducted to measure the degradation rate of the hydrogel over different time periods, as shown in Figure 8. The weight ratio of different components in the hydrogel will significantly impact its breakdown. Hydrogels with different compositions had a degradability percentage ranging from 5.2 to 8.9%. HEC is a biodegradable, biocompatible, naturally occurring polysaccharide derivative of cellulose, which contains three reactive hydroxyl groups in each unit [45]. When exposed to water, these groups alter along with the hydroxyethyl group (CH_2_CH_2_OH), making the polymer soluble for a period of time [46]. Our study also found a difference in the degree of biodegradation between various HEC concentrations. This could be caused by forming functional groups, resulting in many free radicals, which play an essential role in the polymerization reaction and thus contribute to a slower degradation rate.

### 2.10. Structural Characteristics of Hydrogels

The structural parameters of the synthesized hydrogels were determined, including a measure of the degree to which the polymer has been crosslinked (Mc), a measure of polymer volume fraction V_2,s_ (the amount of fluid absorbed by the network and retained by it), the solvent interaction parameter (χ), the number of repeating units between crosslinks (N), and the diffusion coefficient (D) [47]. The values of several structural characteristics are displayed in Table 3. Hydrogels require these values to determine their compatibility with the polymers, thus determining their maximal absorption and holding capacity. The values of V_2,S_ increased with increasing EGDMA concentration, suggesting tighter and stiffer gel structures [48]. Additionally, Mc and N declined with increasing EGDMA concentration. In this case, the crosslinking density increased, accompanied by an increase in EGDMA, which resulted in a decrease in Mc and N. An increase in V_2,s_ indicates an increase in polymer volume.

### 2.11. Swelling Behavior of Hydrogels

**Figure 9:**
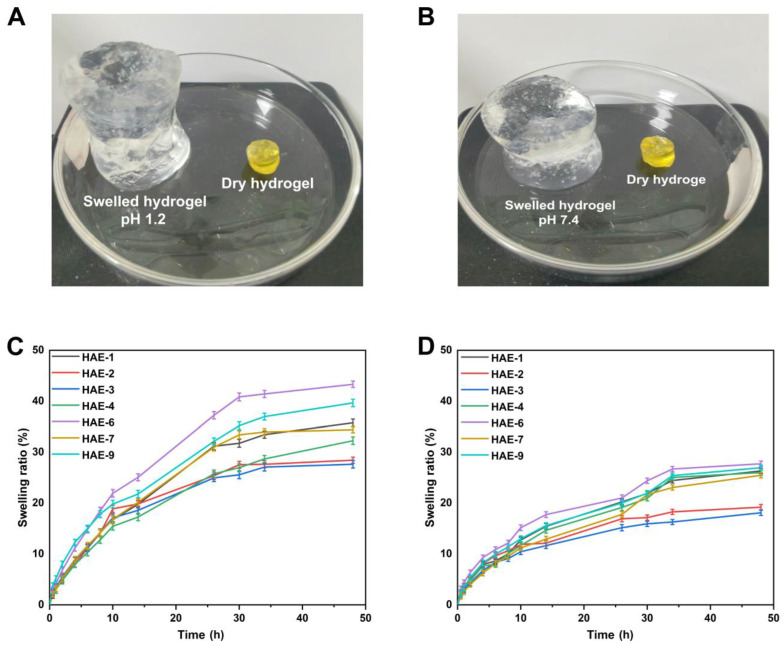
The physical characteristics of hydrogels when swollen at pH 1.2 (**A**) and 7.4 (**B**). The time-dependent swelling graphs of hydrogels at pH 1.2 (**C**) and pH 7.4 (**D**).

Hydrogels containing varying amounts of polymer/monomer and crosslinker concentrations (HEC, AMPS, and EGDMA) were prepared to investigate the effect of these components on swelling ratios and drug release in various media. The hydrophilicity and crosslinking of all polymers in hydrogel composites cause the composites to swell when soaked in various aqueous fluids [49]. It was found that the hydrogel swelled more at pH = 1.2 but slightly less at pH = 7.4 (Figure 9). Since the water was present in the environment, the hydrogels’ hydroxyl (-OH) functional groups were ionized, resulting in an enhanced swelling effect [50]. A pH of 1.2 resulted in a swelling degree of between 27.63% and 43.33% for the hydrogel. The HAE-6 displayed the highest degree of swelling (43.31%) at pH = 1.2, while the HAE-3 displayed the lowest degree of swelling (27.63%) at pH = 1.2. At pH = 7.4, the degree of swelling ranged from 18.09% to 27.74%. At pH = 7.4, HAE-6 had the most significant swelling (27.73%), while HAE-3 had the least swelling (18.09%). The swelling rate increased as the HEC ratio concentration increased, in agreement with previous findings [51]. Increasing the AMPS concentration in a hydrogel will increase its equilibrium swelling degree since AMPS contains many -CONH_2_ and -SO_3_OH groups. The more these groups ionize, the greater the potential interaction between them and absorbent water molecules [52]. A higher concentration of EGDMA reduced swelling. A higher concentration of EGDMA results in a higher packing density for hydrogels. This, in turn, reduces their porosity. Therefore, the amount of water that can penetrate the hydrogel network is limited, and swelling decreases when the concentration of EGDMA is increased and vice versa.

### 2.12. Release and Kinetic Modelling

**Figure 10:**
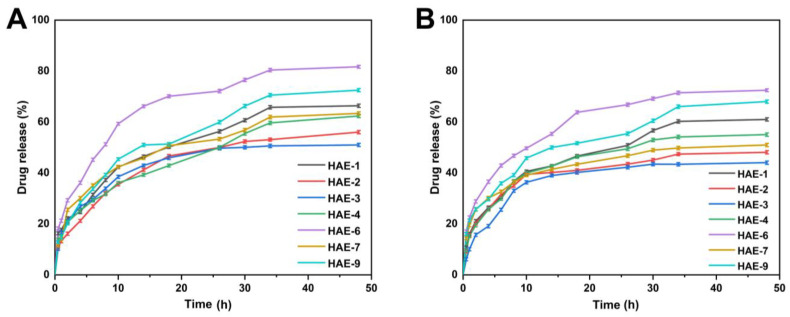
The release of RPA–SD from HEC-*g*-AMPS hydrogels at pH 1.2 (**A**) and pH 7.4 (**B**) over a 48-h time.

Figure 10 displays the range of drug release percentages in the buffer at pH = 1.2, which was between 51.10 and 81.70%. At pH = 1.2, HAE-6 showed the greatest drug release (81.70%), while HAE-3 showed the least (51.10%). Drug release rates ranged from 44.04 to 72.46% in the buffer with pH = 7.4. HAE-6 had the largest drug release (72.46%), whereas HAE-3 had the lowest (44%). The release curve revealed that the release of RPA-SD differed between pH buffers, with the greatest release occurring in the pH = 1.2 buffer. The maximal rate of drug release after 48 h was 81.70%.

The submersion of hydrogel discs in water results in an osmotic pressure gradient, making it possible for water molecules to penetrate the polymer network. In response to water exposure, the hydrogel discs expand, allowing channels to open and releasing the pharmaceutical payload. The model that best matches the release data was chosen based on the regression coefficient value near 1. Table 4 shows regression coefficients (r) for samples with varying HEC concentrations (HAE-1, HAE-7, and HAE-9). As a result of their regression coefficient values being more significant than zero order and first order, these samples demonstrated Higuchi release kinetics. With variations in EGDMA crosslinker amounts, samples (HAE-1, HAE-2, and HAE-3) also exhibited regression coefficient values close to 1, corresponding to Higuchi release kinetics. Also, the Higuchi model regression coefficients (r) for all samples (SAE-1, SAE-4, and SAE-6) with various concentrations of AMPS indicate that diffusion-controlled drug release occurs. RPA-SD-loaded samples (HAE-1, HAE-2, HAE-3, HAE-4, HAE-6, HAE-7, and HAE-9) exhibited non-Fickian diffusion according to the Korsmeyer-Peppas model, with release exponents (n) between 0.5 and 1 [53].

### 2.13. Antioxidant Effects of Hydrogels

**Figure 11:**
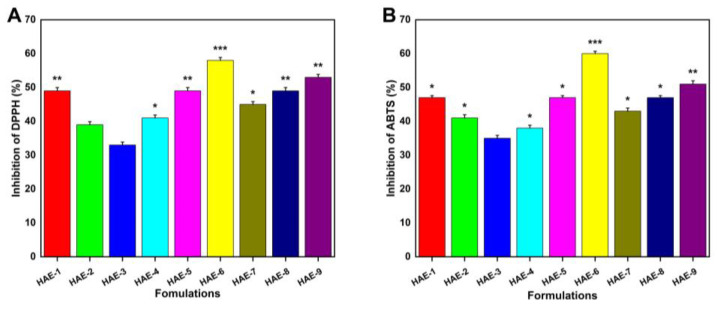
Hydrogel antioxidant activity was measured with DPPH (**A**) and ABTS (**B**). Here, * represent *p* < 0.05, ** *p* < 0.01, and *** *p* < 0.001.

Figure 11 demonstrates the hydrogels’ antioxidant activity by measuring their ability to scavenge ABTS and DPPH radicals. Four formulations (HAE-6, HAE-9, HAE-1, and HAE-7) exhibited greater antioxidant activity than the others. This investigation made use of a well-known medicinal herb called *Radix Paeonia Alba*, which has been shown to have anti-inflammatory and immunomodulatory effects [54]. Different proportions of hydrogels loaded with RPA-SD exhibited potent antioxidant effects, as demonstrated by our experimental findings.

### 2.14. Antibacterial Properties Evaluation

**Figure 12:**
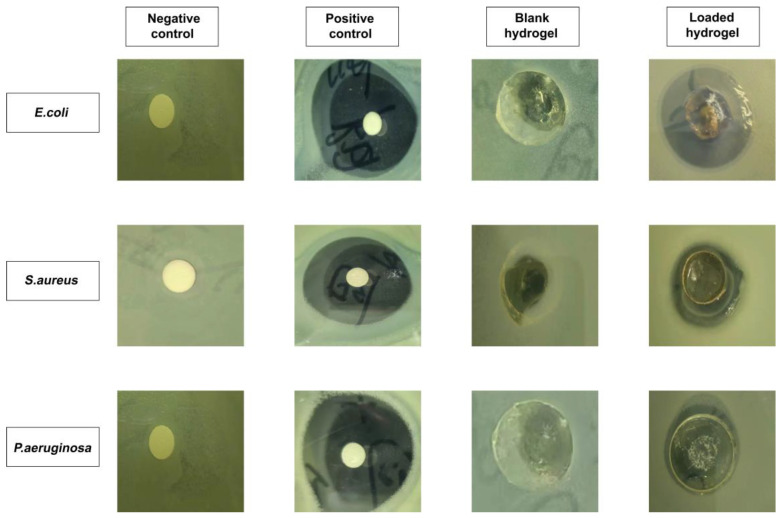
The ZOI of negative control, positive control (cefepime), blank and RPA-SD-loaded hydrogels against different bacteria.

Figure 12 depicts the results of an investigation of antibacterial activity against Gram-positive and Gram-negative microorganisms. It was demonstrated that there was no clear formation of a zone of inhibition (ZOI) observed in the negative control and blank hydrogel group. On the other hand, obvious zones were visible in the positive control (i.e., 35 mm, 34 mm, and 32 mm) and RPA-SD loaded hydrogel disc (i.e., 29 mm, 16 mm, and 12 mm) against *E. coli*, *S. aureus*, and *P. aeruginosa*, respectively [55]. The ZOI of the RPA-SD loaded hydrogel against *Escherichia coli*, *Staphylococcus aureus*, and *Pseudomonas aeruginosa* was 82.85%, 47.05, and 37.50%, respectively.

## 3. Materials and Methods

### 3.1. Materials

Ethylene glycol dimethacrylate (EGDMA; MW: 198.22 g/moL), 2-Acrylamido -2-methyl-1-propanesulfonic acid (AMPS; MW: 207.25 g/moL), and ammonium persulfate (APS) were obtained from Sigma-Aldrich, Saint Louis, MO, USA. Sodium bisulfite (SHS) was purchased from Shanghai Aladdin biochemical technology, China. ABTS, DPPH, and Cefepime hydrochloride were purchased from Meilune biological company (Dalian, China). Hydroxyethyl cellulose (HEC; MW: 736.7 g/moL) was obtained from Meilune biological company (China). *Radix Paeonia Alba* was obtained from Jiangxi Jiangzhong Traditional Chinese Medicine Co., Ltd., (Nanchang, China) and authenticated by Prof. Deng Kezhong (School of Pharmacy, Jiangxi University of Chinese Medicine; voucher number: J20221012).

*Escherichia coli* (*E. coli*: ATCC25922HBJZ087), *Staphylococcus aureus* (*S. aureus*: ATCC25923HBJZ005), and *Pseudomonas aeruginosa* (*P. aeruginosa*: ATCC27853HBJZ017) bacterial strains were procured from Qingdao Hope Biotechnology, Co., Ltd., Qingdao, China.

### 3.2. Development of Radix Paeonia Alba Amorphous Solid Dispersion (RPA–SD)

RPA 50 g was added to 6000 mL of ultra-purified water in an electrical heating sleeve device and heated to a specific temperature. After that, it was soaked for 30 min. A heating reflux method was then used to extract RPA extract, which was then filtered through mesh screens. The filtered extract of RPA was concentrated using a rotating vacuum evaporator from Xiamen Jingyi Xingye Technology Co., Ltd. (Xiamen, China) The extract was then frozen at −20 °C for 24 h. The sample was then transferred to a lyophilizer (Ningbo Xinzhi Biotechnology Co., Ltd., Ningbo, China) and freeze-dried for three days [56].

#### Identification of Compounds in RPA-SD Using the UHPLC-Q-TOF-MS Method 

A standard UHPLC analysis was performed using an ACQUITY UPLC^®^ BEH C18 column (2.1 × 100 mm, 1.8 μm) at 30 °C [57]. Elution was performed using a gradient of A (0.1% formic acid in distilled water) and B (Acetonitrile) with a flow rate of 0.25 mL/min as follows: 0~18 min 5%~40% B; 18~55 min 40%~95% B; 55~57 min 95%~95% B; 57~57.1 min 95%~5% B; 57.1~60 min 5%~5% B. The samples were injected at a volume of 1 µL. Waters XeVO G2-S QT mass spectrometers equipped with water electrospray ionization interfaces (Waters, Milford, MA, USA) were used in the present study to collect mass spectrometer data. Time of flight mass spectrometry (TOF/MS) optimal conditions were established by adjusting the following parameters: the desolvation gas flow rate was 600 L/h; the desolvation temperature was 550 °C; the capillary voltage was 4.5–5 kV; the source temperature was 500 °C; the conical hole voltage was ±100 V and the cone gas flow was 50 L/h. This mass spectrometer is configured to scan over a range of 100~1500 *m*/*z* at a collision energy of ±30 eV. A MassLynx 4.1 software program was used to determine the precise mass of each component and fragment ion.

### 3.3. Preparation of HEC-g-AMPS Hydrogels

**Figure 13:**
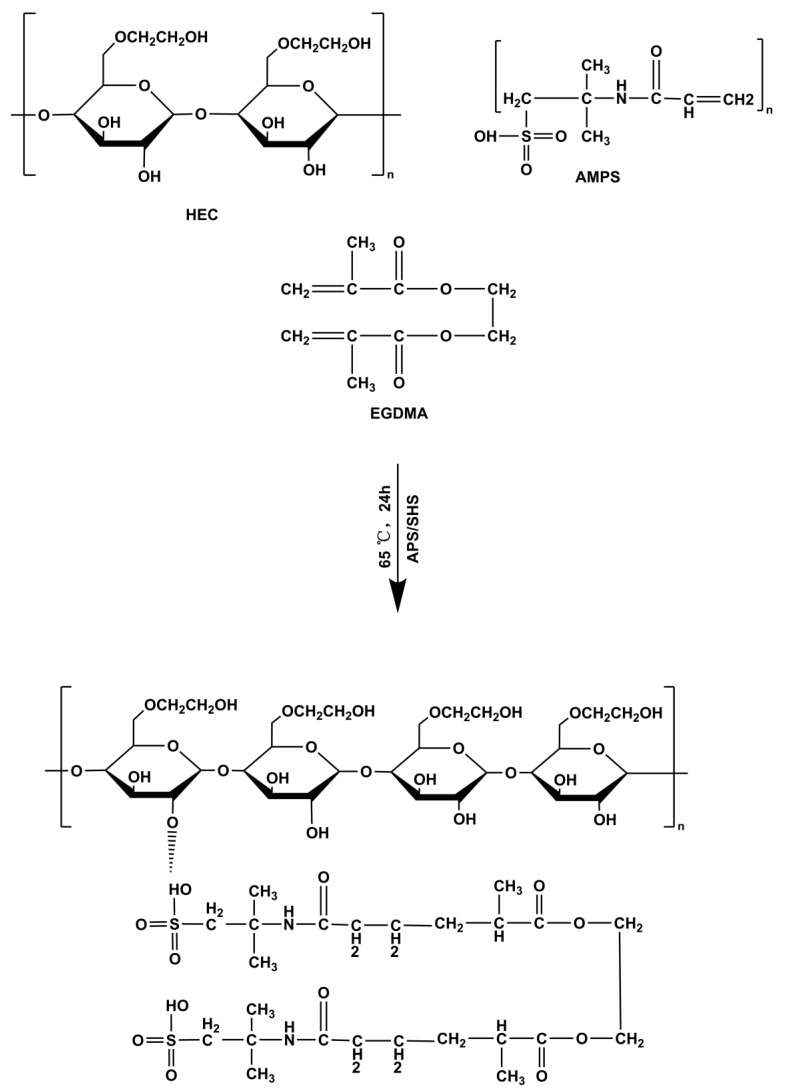
Proposed crosslinked chemical structure of HEC-*g*-AMPS hydrogels.

The free radical polymerization technique was utilized to prepare hydrogels with slight modifications [58]. Specifically, for each hydrogel formulation, the number of specified ingredients, such as HEC, AMPS, APS/SHS, and EGDMA, was measured and placed into separate glass bottles, as outlined in Table 5, which was slightly modified from our previous study by replacing sodium alginate with hydroxyethyl cellulose [59]. After the water was added to each vial with a label, the solution was continuously stirred until it became clear. The HEC polymer was stirred continuously at 40 °C until fully dissolved. Once the labeled solutions were prepared, they were mixed by their defined mixing sequence. Once APS and SHS had been added to AMPS, they were mixed evenly before being added to HEC to ensure homogeneity. Afterward, the homogeneous mixture was ultrasonically mixed, and nitrogen bubbled for 30 min. In the end, EGDMA was added to the mixture dropwise. Once the final solution was prepared, it was transferred to a water bath at 50 °C. Gradually, the temperature was raised to 65 °C. Following a 24-h gelling process, the hydrogels were cut into 8 mm discs and vacuum-dried to obtain dried hydrogels. A proposed structural diagram of HEC-*g*-AMPS is depicted in Figure 13.

#### RPA–SD Loading into Hydrogels

RPA-SD was loaded into hydrogels using the swelling-diffusion method [60]. Briefly, RPA-SD solution (1% *w*/*v*) was prepared in phosphate buffer at pH 7.4. Then, dried discs of hydrogels were placed in the solution and stirred for approximately 48 h. After the solution was removed, the discs were rinsed with distilled water. Hydrogel discs were dried at room temperature and then incubated at 40 °C. The amount of drug within the hydrogel was calculated by subtracting the weight of the initially unloaded hydrogel from the weight of the RPA–SD–loaded hydrogel.

(1) RPA−SD loading=RPA−SD loaded hydrogel−Unloaded hydrogel

### 3.4. In Vitro Solid-State Characterization

#### 3.4.1. Fourier-Transform Infrared (FTIR) Study

Attenuated total reflectance (ATR) FTIR spectra were collected with a Spectrum Two FTIR spectrometer (Perkin Elmer, Beaconsfield, UK) with a range of 400–4000 cm^−1^. After the samples were crushed into the desired size, they were placed in the sample compartment, and all spectra were taken directly from the powder. A comparison was made between the spectra of the developed dosage form and their reactants [61].

#### 3.4.2. Thermal Studies 

Data on thermal analysis were collected using a TG/DTA6300TG thermal analysis system (SII Nano, Tokyo, Japan). The samples (0.5–5 mg) were analyzed at temperatures ranging from 30 °C to 600 °C under a dynamic nitrogen atmosphere and a purge gas flow rate of 10 mL/min to determine the samples’ weight changes as a function of temperature [62].

Differential scanning calorimetry (DSC) was performed using a DSC system (Perkin Elmer, UK). Samples of 0.5 to 5.0 mg were weighed and sealed in aluminum pans before analysis. The analysis was conducted at 30 to 350 °C with a continuous heating rate of 10 °C/min and nitrogen gas purging at 10 mL/min [63].

#### 3.4.3. X-ray Diffraction (XRD) Studies

The crystallinity of the reactants and the prepared hydrogel formulations was determined using an X-ray diffractometer (TD-3500 X-ray diffractometer, Tongdatek, Dandong, China) with CuKα irradiations at a voltage of 30 kV and a current of 20 mA. The scanning was carried out in the range of 2-theta degrees of 10–60° at 2°/min. The samples measured included HEC, RPA-SD loaded and unloaded formulations [64].

#### 3.4.4. Morphological Characteristics of Hydrogels

Scanning electron microscope (Quanta 250, FEI, Eindhoven, The Netherlands) was employed to determine the morphological properties, such as porosity, roughness and other characteristics. The samples were placed on an aluminum stub, coated with gold, and then observed under a 15 kV accelerated current [65].

#### 3.4.5. Tensile Behavior of Hydrogels 

The hydrogels were tested for their tensile strength (TS) and elongation at break (EAB) using a TA.XT analyzer (Stable Micro Systems, Godalming, UK) equipped with a cylindrical probe. The following formulas are used for the calculation of the mechanical properties [66].

(2) $$TS=\frac{Fm}{Th}$$

(3) $$EAB=\frac{\sqrt{D^{2}+R^{2}}}{R}-1$$

Fm is the force the probe applies, while Th is hydrogel thickness. D is the displacement, and R is the radius of the plate.

#### 3.4.6. Sol-Gel Fraction Investigation

Sol-gel study was performed to determine the crosslinked and uncrosslinked fractions of the synthesized hydrogels. Generally, the sol fraction is part of the hydrogel formulation, which remains uncrosslinked due to the unavailability of many functional groups in the reaction mixture for reaction with it. This happens if the increased amount of any reactant is used excessively in the formulation. While the gel fraction represents the crosslinked part of the hydrogel after successful crosslinking. Usually, the gel fraction is stronger if a higher quantity of the crosslinker or the polymers are used to prepare the hydrogel. Briefly, sol-gel analysis was carried out by placing a specified amount of hydrogels in purified water at 85 °C in a round bottom flask fitted with a condenser for 10 h. After that, the hydrogels were taken out and dried, and their weights were taken. The following equations were used to calculate the sol and gel fractions.

(4) $$Sol\;fraction\;\%=\frac{R1-R2}{R2}\times 100$$

(5) Gel fraction=100−sol fraction

whereas, R1 and R2 are the weights of the hydrogels before and after extraction, respectively.

#### 3.4.7. Porosity of Hydrogels

The porosity was determined using a solvent-replacement methodology. Briefly, the dried preweighed hydrogel discs (B1) were immersed in the absolute ethanol for 5 days and then taken out and blotted with filter paper to remove the surface attached to water and then its weight (B2) and dimensions were measured, and the following equation was used to determine it [67].

(6) $$Porosity\;percentage\left(\%\right)=\frac{B2-B1}{\rho v}\times 100$$

whereas, ρ is ethanol’s density and v is hydrogel’s volume.

#### 3.4.8. Equilibrium Swelling Ratio (ESR)

The swelling behavior of the fabricated hydrogels was analyzed in pH 7.4 and pH 1.2 phosphate buffers. Briefly, hydrogel discs with predetermined weight were slowly placed in the respective buffers and taken out at predefined time intervals, cleaned the surfaced-attached water with a filter paper, weighed and immersed in the same buffer solution again till equilibrium was reached. The given equation was used for its calculation [68].

(7) $$ESR=\frac{Y_{t}-Y_{i}}{Y_{i}}\times 100$$

whereas, Y_t_ shows the weight of hydrogel at time t and ‘Y_i_’ represents the initial hydrogel weight.

#### 3.4.9. In Vitro Release Study and Kinetic Data Modelling

Drug release studies of the synthesized hydrogels were conducted at both pH 1.2 and pH 7.4 to evaluate pH-dependent and controlled drug release [69]. Drug-containing hydrogel discs were immersed in 900 mL phosphate buffer solution in a USP dissolution device type II at 37 °C and 50 rpm. Throughout the experiment, samples were taken at predetermined intervals, and the medium was always replaced with fresh medium. A UV-Vis spectrophotometer (T6 New Century; Beijing GM, Beijing, China) was used to analyze the samples in triplicate at 265 nm (maximum absorption wavelength) [70].

(8) $$Drug\;release\%=\frac{\left(Amount\;of\;released\;drug\right)}{\left(Amount\;of\;loaded\;drug\right)}\times 100$$

Drug release is affected by many factors such as drug nature, matrix type, the hydrogel’s swelling potential, the polymeric chains’ strength, media pH, etc. Dissolution data were analyzed using mathematical models to determine how drugs are released from cross-linked hydrogels. This was accomplished using the DD solver. We evaluated the drug release pattern from polymeric networks of hydrogels by applying kinetic models such as zero-order, first-order, Higuchi, and Korsmeyer–Peppas models.

(9) Zero−order kinetics Ft=K0t

In this case, Ft refers to the drug released at time t, and K0 corresponds to the apparent zero-order rate constant of release.

(10) $$First-order\;kinetics\;ln\left(1-F\right)=-K1t$$

F denotes the drug release at time t, whereas k1 denotes the first-order release rate constant.

(11) $$Higuchi\;model\;F={K2t}^{\frac{1}{2}}$$

where, K2 represents Higuchi’s constant, while F represents the drug’s release rate at time t.

(12) $$Korsmeyer-Peppas\;model\;\frac{Mt}{M¥}={K3t}^{n}$$

In equilibrium, the amount of water absorbed is denoted by M∞, and at time t, it is denoted by Mt. K3 represents a constant that considers the geometric and structural characteristics of the gels, and n represents the release exponent for the gels.

### 3.5. Structural Parameters Affecting Hydrogel Networks

The structure and properties of swollen hydrogels can be determined based on several important factors [71].

#### 3.5.1. Diffusion Coefficient (D)

It can be determined by the given equation.

(13) $$D=\pi {\left(\frac{h\times \theta }{4\times qeq}\right)}^{2}$$

while q*e*q describes equilibrium swelling, θ expresses linear slopes of swelling curves, and h indicates the disc thickness.

#### 3.5.2. Volume Fraction of Polymer (V_2,s_)

The formula for calculating it is as follows.

(14) $$V_{2,s}=\frac{1}{Veq}$$

#### 3.5.3. Distribution of Molecular Weights between Crosslinks (Mc)

Mc measures the crosslinking of polymer networks. The equation below can be used to calculate it.

(15) $$Mc=\frac{dpVs\left(V^{\frac{1}{3}}{}_{2,s}-V_{2,s}/2\right)}{\ln\left(1-V_{2,s}\right)+V_{2,s}+\chi V_{2,s}^{2}}$$

Here, dp represents the density of the polymer, Vs denotes the volume of the solvent, and χ is the Flory-Huggins polymer-solvent interaction parameter.

#### 3.5.4. Solvent Interaction Factor (χ)

It can be computed by the given equation.

(16) $$\chi =\frac{\ln\left(1-V_{2,s}\right)+V_{2,s}}{V_{2,s}^{2}}$$

whereas, “V_2,s_” indicates volume fraction.

#### 3.5.5. Crosslink Repeating Units (N)

Crosslink repeating units were calculated using the following equation.

(17) $$N=\frac{2Mc}{Mr}$$

Mr represents the molar mass of repeating units and is calculated as follows.

(18) $$Mr=\frac{mHECMHEC+mAMPSMAMPS+mEGDMAMEGDMA}{mHEC+mAMPS+mEGDMA}$$

whereas, m represents mass and M indicates the molar mass of the ingredients used in the hydrogel preparation.

### 3.6. In Vitro Biodegradation of Hydrogels

The biodegradation study of the hydrogel formulations was conducted in phosphate buffer (pH 7.4) and 37 ± 0.5 °C. Briefly, hydrogel discs were placed in the buffer solution for an appropriate time, taken out, dried, and then placed back in the solution, and the weight changes were continuously recorded. The degradation rate of hydrogels was determined using the following equation [72].

(19) $$D=\frac{U1-U2}{U1}$$

while D stands for degradation, U1 stands for dry sample weight, and U2 stands for sample weight after immersion at some time (t).

### 3.7. Potential Antioxidant Effects of the Hydrogels

#### 3.7.1. DPPH Assay

The antioxidant potential of hydrogels was measured using a DPPH free radical scavenging test [73]. The samples were mixed with methanol and left in the dark for 24 h at room temperature. The hydrogel extract solution was then combined with 0.1 mM DPPH-methanol solution. After shaking the mixture thoroughly, it was placed in a cool, dark location for half an hour. A UV-Vis spectrophotometer was used at the end of the process to measure the absorbance at 517 nm of the solution. The following equation was used to calculate the level of DPPH scavenging activity (DPPH%).

(20) $$DPPH\left(\%\right)=\frac{E0-E}{E0}\times 100$$

where E0 is the control sample absorbance, and E is the test sample absorbance.

#### 3.7.2. ABTS Assay

Hydrogels loaded with RPA-SD were tested for their radical scavenging activity using the ABTS assay [74]. The radicalization of ABTS was caused by incubating a 1:1 mixture of ABTS (7.4 mM) and potassium persulfate (2.4 mM) at ambient temperature for a night. The hydrogels were then combined with ABTS solution and stored at 37 °C for 30 min. The absorbance was checked at a wavelength of 730 nm. The following equation was used to determine how effective hydrogels are against ABTS as a free radical scavenger.

(21) $$ABTS\;scavenging\;effect\left(\%\right)=\frac{B0-B1}{B0}\times 100$$

The absorbance of the ABTS standard is denoted by B0, while that of the samples is denoted by B1.

### 3.8. Antibacterial Effects of Hydrogels

Nutritional agar was produced by dissolving agar growth media in distilled water and autoclaving the solution for 30 min at 121 °C and 15 psi at 1 atmosphere of pressure. The liquid agar medium was transferred to petri dishes and then cooled to room temperature. This process solidified the media. The 24-h-grown strains of *Pseudomonas aeruginosa*, *Staphylococcus aureus*, and *Escherichia coli* were swabbed and plated on Petri dishes. They were split into the following four plates: control (unloaded hydrogels), test sample (RPA-SD-loaded hydrogels), positive control (cefepime, 1 mg/mL solution), and negative control (empty tissue). The inhibitory zone was established after incubating the plates at 37 °C for 24 h [75].

(22) $$Percentage\;inhibition=\frac{Zone\;of\;inhibition\;of\;test\;sample\left(mm\right)}{Zone\;of\;inhibition\;of\;standard\;drug\left(mm\right)}\times 100$$

### 3.9. Statistical Data Analysis

Statistical information was summarized using a mean and standard deviation format. We utilized a two-way ANOVA followed by Tukey’s posthoc test to compare the data in each set to find statistically significant differences. Statistical significance was determined by calculating the corresponding *p*-value, which was given as follows: * *p* < 0.05, ** *p* < 0.01, and *** *p* < 0.001.

## 4. Conclusions

In this study, an amorphous solid dispersion of *Radix Paeonia Alba* was developed and studied using UHPLC-Q-TOF-MS, resulting in the identification of 43 compounds (mostly flavonoids, nucleosides, terpenoids and phenolic acids). The controlled release hydrogel carrier system developed for RPA-SD delivery into the body via the oral route was developed by grafting 2-acrylamido-2-methylpropane sulfonic acid (AMPS) onto the backbone of the semi-synthetic polymer hydroxyethyl cellulose (HEC). According to FTIR, XRD, TGA, and DSC analysis, hydrogel networks were formed, and the drug was successfully loaded (RPA-SD), while SEM studies indicated a porous structure. Higher polymer ratios and monomer concentrations led to greater swelling (ESR of 43.33% at pH 1.2 and 27.74% at pH 7.4), longer drug release durations (81.7% at pH 1.2 and 72.4% at pH 7.4), and enhanced mechanical characteristics. In addition, the hydrogels exhibited excellent porosity (84.15%) and biodegradability (8.9% per week). In DPPH and ABTS experiments, hydrogels also showed good antioxidant activity. Furthermore, the hydrogels exhibited significant antibacterial effects against both Gram-positive (*E. coli*, ZOI 29 mm; *S. aureus*, ZOI 16 mm) and Gram-negative (*P. aeruginosa*, ZOI 12 mm) bacteria. HAE-6 having a higher concentration of AMPS was the optimized formulation and showed better performance compared to other formulations in the study. In summary, HEC-*g*-AMPS hydrogels can be used instead of traditional dosage forms to deliver solid dispersions of medicinal plants and hydrophilic drugs in a controlled manner.

## Figures and Tables

**Table 1:** Compounds identified in the RPA-SD using UHPLC-Q-TOF-MS.

Peak No.	Retention Time/min	Compounds	Formula	Experimental *m*/*z*	Theoretical *m*/*z*	Error (ppm)	Mode
1	1.26	Sucrose	C_12_H_22_O_11_	341.10921	341.10894	0.8	−
2	3.41	Desbenzoyl paeoniflorin	C_16_H_24_O_10_	375.12996	375.12967	0.5	−
3	4.16	6-O-galloylglucose	C_13_H_16_O_10_	331.06762	331.06707	1.7	−
4	4.35	Pyrogallol	C_6_H_6_O_3_	127.03939	127.03897	3.3	+
5	4.93	Moutonone-1-O-β-D-glucoside or isomer	C_16_H_24_O_9_	359.13561	359.13476	2.4	−
6	5.45	1’-0-galloylsucrose or isomer	C_19_H_26_O_15_	493.12034	493.11989	0.9	−
7	7.07	Paeonol	C_9_H_10_O_3_	167.07021	167.07027	−0.4	+
8	8.09	Methyl gallate	C_8_H_8_O_5_	185.04433	185.04445	−0.6	+
9	8.30	Mudanpioside F	C_16_H_24_O_8_	343.14057	343.13984	2.1	−
10	10.47	Oxypaeoniflorin	C_23_H_28_O_12_	495.15169	495.1508	1.8	−
11	10.47	Catechin	C_15_H_14_O_6_	289.07331	289.07176	5.4	−
12	10.49	Salicylicacid	C_7_H_6_O_3_	139.03915	139.03897	1.3	+
13	11.97	Paeonoside	C_15_H_20_O_8_	327.10858	327.10854	0.1	−
14	12.84	6-O-β-d-glucosyl pyran-paeoniolide	C_16_H_26_O_9_	361.15151	361.15041	3.1	−
15	13.59	Paeonilactone B	C_10_H_12_O_4_	197.0807	197.08084	−0.7	+
16	14.34	Cortex moutan i	C_23_H_28_O_11_	479.15633	479.15589	0.9	−
17	14.35	Cortex moutan e	C_24_H_30_O_13_	525.16158	525.16137	0.4	−
18	14.36	Lactiflorin	C_23_H_26_O_8_	463.15977	463.15987	−0.2	+
19	14.38	Paeoniflorin	C_23_H_28_O_11_	479.15633	479.15589	0.9	−
20	14.42	Albiflorin	C_23_H_28_O_11_	479.15633	479.15589	0.9	−
21	14.42	Agnuside	C_22_H_26_O_11_	465.12014	465.14024	−1.2	−
22	17.44	Galloylpaeoniflorin or isomer	C_30_H_32_O_15_	631.16802	631.16684	1.9	−
23	17.56	Galloylpaeoniflorin	C_30_H_32_O_15_	631.16802	631.16684	1.9	−
24	17.8	Benzoicacid	C_7_H_6_O_2_	123.04448	123.04406	3.4	+
25	18.02	1,2,3,4,6-O-pentagalloylglu-cose	C_41_H_32_O_26_	939.11402	939.11091	3.3	−
26	20.11	Mudanpioside D	C_24_H_30_O_12_	509.16701	509.16645	1.1	−
27	21.17	3’,6’-di-O-galloylpaeoniflorin	C_37_H_36_O_19_	783.18044	783.1778	3.4	−
28	21.89	Benzoyloxypaeoniflorin	C_30_H_32_O_13_	599.17816	599.17702	1.9	−
29	21.89	Mudanpioside H	C_30_H_32_O_14_	615.17351	615.17193	2.6	−
30	21.99	Mudanpioside C	C_30_H_32_O_13_	599.17816	599.17702	1.9	−
31	25.51	Benzoylpaeoniflorin	C_30_H_32_O_12_	583.18289	583.1821	1.4	−
32	25.59	Mudanpioside J	C_31_H_34_O_14_	629.18919	629.18758	2.6	−
33	25.59	Mudanpioside B	C_31_H_34_O_14_	629.18919	629.18758	2.6	−
34	25.88	Benzoylpaeoniflorin	C_30_H_32_O_12_	583.18289	583.1821	1.4	−
35	28.49	Formononetin	C_16_H_12_O_4_	269.08087	269.08084	0.1	+
36	29.49	Palbinone	C_22_H_30_O_4_	357.20833	357.20713	3.6	−
37	35.60	30-norheder-agenin	C_29_H_44_O_4_	455.31751	455.31668	1.8	−
38	38.24	Betulinic acid	C_30_H_46_O_3_	455.35235	455.35197	0.8	+
39	41.42	Dibutylphthalate	C_16_H_22_O_4_	279.15918	279.15909	0.3	+
40	43.92	Hederagenol	C_30_H_48_O_4_	473.36044	473.36254	−4.4	+
41	44.03	2,3-hydroxybetulinicacid	C_30_H_48_O_4_	473.36044	473.36254	−4.4	+
42	50.28	Palmitic acid	C_16_H_32_O_2_	255.23399	255.23295	4.1	−
43	54.33	Ethyl palmitate	C_18_H_36_O_2_	283.26519	283.26425	3.3	−

**Table 2:** Mechanical characteristics of HEC-*g*-AMPS hydrogels.

F. Codes	Thickness (mm)	TS (N/m^2^)	EAB (%)	RPA-SD Loaded/1 g Hydrogel (g)
HAE-1	1.15	0.677	36.9	0.488
HAE-2	1.27	0.824	69.7	0.411
HAE-3	1.26	1.124	75.8	0.356
HAE-4	0.98	0.716	55.7	0.465
HAE-5	1.15	0.677	36.9	0.488
HAE-6	1.26	0.414	31.5	0.571
HAE-7	1.18	0.635	32.5	0.481
HAE-8	1.15	0.677	36.9	0.488
HAE-9	1.19	0.713	60.8	0.491

**Table 3:** The Flory-Huggins network parameters for HEC-g-AMPS hydrogels.

F. Codes	V_2,s_	χ	M_c_	M_r_	N	D × 10^−5^ (cm^2^ s^−1^)
HAE-1	0.020	0.506	4080.781	219.380	37.202	0.017
HAE-2	0.029	0.509	3971.142	218.883	34.640	0.016
HAE-3	0.033	0.511	1013.333	218.409	9.279	0.023
HAE-4	0.027	0.509	2203.846	227.000	19.417	0.024
HAE-5	0.020	0.506	4080.781	219.380	37.202	0.017
HAE-6	0.018	0.506	5730.769	215.965	53.071	0.011
HAE-7	0.024	0.508	3285.714	214.413	30.648	0.019
HAE-8	0.020	0.506	4080.781	219.380	37.202	0.017
HAE-9	0.022	0.507	1222.727	224.254	10.904	0.016

**Table 4:** RPA-SD release kinetics from HEC-*g*-AMPS hydrogel at different pH levels based on different compositions of hydrogels.

F. Codes	pH	Zero Order		First Order		Higuchi Model		Korsmeyer-Peppas Model	
		K_o_ (h^−1^)	r^2^	K_1_ (h^−1^)	r^2^	K_2_ (h^−1^)	r^2^	r^2^	*n*
HAE-1	1.2	1.837	0.9446	0.032	0.9888	10.406	0.9879	0.9878	0.527
	7.4	1.404	0.9105	0.021	0.9539	8.155	0.9825	0.9861	0.426
HAE-2	1.2	1.484	0.9604	0.022	0.9878	8.398	0.9967	0.9969	0.521
	7.4	0.920	0.9483	0.012	0.9660	5.309	0.9979	0.9983	0.428
HAE-3	1.2	1.036	0.9232	0.014	0.9504	5.989	0.9882	0.9906	0.439
	7.4	0.824	0.9226	0.010	0.9421	4.821	0.9913	0.9962	0.382
HAE-4	1.2	1.243	0.9509	0.017	0.9760	7.109	0.9963	0.9963	0.473
	7.4	0.972	0.9469	0.012	0.9651	5.616	0.9981	0.9992	0.419
HAE-5	1.2	1.837	0.9446	0.032	0.9888	10.406	0.9879	0.9878	0.527
	7.4	1.404	0.9105	0.021	0.9539	8.155	0.9825	0.9861	0.426
HAE-6	1.2	1.740	0.9540	0.029	0.9906	9.823	0.9921	0.9922	0.539
	7.4	1.393	0.9672	0.020	0.9899	7.867	0.9964	0.9971	0.528
HAE-7	1.2	1.553	0.8964	0.024	0.9209	8.851	0.9376	0.9377	0.485
	7.4	1.039	0.9565	0.014	0.9733	5.953	0.9989	0.9990	0.455
HAE-8	1.2	1.837	0.9446	0.032	0.9888	10.406	0.9879	0.9878	0.527
	7.4	1.404	0.9105	0.021	0.9539	8.155	0.9825	0.9861	0.426
HAE-9	1.2	1.440	0.9542	0.022	0.9814	8.230	0.9979	0.9980	0.472
	7.4	1.178	0.9566	0.016	0.9780	0.730	0.9985	0.9985	0.473

**Table 5:** Chemical composition of HEC-*g*-AMPS hydrogels.

Formulation Codes	HEC (g)	AMPS (g)	APS/SHS (g)	EGDMA (g)
HAE-1	0.5	20	0.3/0.3	**0.5**
HAE-2	0.5	20	0.3/0.3	**1**
HAE-3	0.5	20	0.3/0.3	**1.5**
HAE-4	0.5	**12**	0.3/0.3	0.5
HAE-5	0.5	**20**	0.3/0.3	0.5
HAE-6	0.5	**28**	0.3/0.3	0.5
HAE-7	**0.3**	20	0.3/0.3	0.5
HAE-8	**0.5**	20	0.3/0.3	0.5
HAE-9	**0.7**	20	0.3/0.3	0.5

The bold text implies compositions with higher feeding quantities.

## Data Availability

All the data are contained within the article.

## Sample Availability

Not available.

## References

[1] Byeon J.C., Ahn J.B., Jang W.S., Lee S.-E., Choi J.-S., Park J.-S. (2019). Recent formulation approaches to oral delivery of herbal medicines. J. Pharm. Investig..

[2] Luo L., Jiang J., Wang C., Fitzgerald M., Hu W., Zhou Y., Zhang H., Chen S. (2020). Analysis on herbal medicines utilized for treatment of COVID-19. Acta Pharm. Sin. B.

[3] Canarini A., Kaiser C., Merchant A., Richter A., Wanek W. (2019). Root exudation of primary metabolites: Mechanisms and their roles in plant responses to environmental stimuli. Front. Plant Sci..

[4] Erb M., Kliebenstein D.J. (2020). Plant secondary metabolites as defenses, regulators, and primary metabolites: The blurred functional trichotomy. Plant Physiol..

[5] Elfawal M.A., Towler M.J., Reich N.G., Weathers P.J., Rich S.M. (2015). Dried whole-plant Artemisia annua slows evolution of malaria drug resistance and overcomes resistance to artemisinin. Proc. Natl. Acad. Sci. USA.

[6] Li J.W.-H., Vederas J.C. (2009). Drug discovery and natural products: End of an era or an endless frontier?. Science.

[7] Wagner H., Ulrich-Merzenich G. (2009). Synergy research: Approaching a new generation of phytopharmaceuticals. Phytomedicine.

[8] Weathers P.J., Arsenault P.R., Covello P.S., McMickle A., Teoh K.H., Reed D.W. (2011). Artemisinin production in Artemisia annua: Studies in planta and results of a novel delivery method for treating malaria and other neglected diseases. Phytochem. Rev..

[9] Jürgenliemk G., Nahrstedt A. (2003). Dissolution, solubility and cooperativity of phenolic compounds from *Hypericum perforatum* L. in aqueous systems. Die Pharm.-Int. J. Pharm. Sci..

[10] Zhao Q., Luan X., Zheng M., Tian X.-H., Zhao J., Zhang W.-D., Ma B.-L. (2020). Synergistic mechanisms of constituents in herbal extracts during intestinal absorption: Focus on natural occurring nanoparticles. Pharmaceutics.

[11] Loureiro Damasceno J.P., Silva da Rosa H., Silva de Araújo L., Jacometti Cardoso Furtado N.A. (2022). Andrographis paniculata formulations: Impact on diterpene lactone oral bioavailability. Eur. J. Drug Metab. Pharmacokinet..

[12] Tchicaillat-Landou M., Petit J., Gaiani C., Miabangana E.S., Kimbonguila A., Nzikou J.-M., Scher J., Matos L. (2018). Ethnobotanical study of medicinal plants used by traditional healers for the treatment of oxidative stress-related diseases in the Congo Basin. J. Herb. Med..

[13] Williams H.D., Trevaskis N.L., Charman S.A., Shanker R.M., Charman W.N., Pouton C.W., Porter C.J. (2013). Strategies to address low drug solubility in discovery and development. Pharmacol. Rev..

[14] Taylor L.S., Zhang G.G. (2016). Physical chemistry of supersaturated solutions and implications for oral absorption. Adv. Drug Deliv. Rev..

[15] Tan Y.-Q., Chen H.-W., Li J., Wu Q.-J. (2020). Efficacy, Chemical Constituents, and Pharmacological Actions of Radix Paeoniae Rubra and Radix Paeoniae Alba. Front. Pharmacol..

[16] Yang S., Zhang X., Dong Y., Sun G., Jiang A., Li Y. (2021). Cleavage rules of mass spectrometry fragments and rapid identification of chemical components of Radix Paeoniae Alba using UHPLC-Q-TOF-MS. Phytochem. Anal..

[17] Yan B., Shen M., Fang J., Wei D., Qin L. (2018). Advancement in the chemical analysis of Paeoniae Radix (Shaoyao). J. Pharm. Biomed. Anal..

[18] Kesharwani P., Bisht A., Alexander A., Dave V., Sharma S. (2021). Biomedical applications of hydrogels in drug delivery system: An update. J. Drug Deliv. Sci. Technol..

[19] Zhang X., Zhao J., Xie P., Wang S. (2023). Biomedical Applications of Electrets: Recent Advance and Future Perspectives. J. Funct. Biomater..

[20] Bernhard S., Tibbitt M.W. (2021). Supramolecular engineering of hydrogels for drug delivery. Adv. Drug Deliv. Rev..

[21] Tang J., Yi W., Yan J., Chen Z., Fan H., Zaldivar-Silva D., Agüero L., Wang S. (2023). Highly absorbent bio-sponge based on carboxymethyl chitosan/poly-γ-glutamic acid/platelet-rich plasma for hemostasis and wound healing. Int. J. Biol. Macromol..

[22] Jacob S., Nair A.B., Shah J., Sreeharsha N., Gupta S., Shinu P. (2021). Emerging role of hydrogels in drug delivery systems, tissue engineering and wound management. Pharmaceutics.

[23] Onaciu A., Munteanu R.A., Moldovan A.I., Moldovan C.S., Berindan-Neagoe I. (2019). Hydrogels based drug delivery synthesis, characterization and administration. Pharmaceutics.

[24] El Fawal G.F., Abu-Serie M.M., Hassan M.A., Elnouby M.S. (2018). Hydroxyethyl cellulose hydrogel for wound dressing: Fabrication, characterization and in vitro evaluation. Int. J. Biol. Macromol..

[25] Liu X., Chen T., Liu L., Li G. (2006). Electrochemical characteristics of heme proteins in hydroxyethylcellulose film. Sens. Actuators B Chem..

[26] Cerrato A., Cavaliere C., Montone C.M., Piovesana S. (2023). New hydrophilic material based on hydrogel polymer for the selective enrichment of intact glycopeptides from serum protein digests. Anal. Chim. Acta.

[27] Biglione C., Neumann-Tran T.M.P., Kanwal S., Klinger D. (2021). Amphiphilic micro- and nanogels: Combining properties from internal hydrogel networks, solid particles, and micellar aggregates. J. Polym. Sci..

[28] Kong M., Liu H.-H., Wu J., Shen M.-Q., Wang Z.-G., Duan S.-M., Zhang Y.-B., Zhu H., Li S.-L. (2018). Effects of sulfur-fumigation on the pharmacokinetics, metabolites and analgesic activity of Radix Paeoniae Alba. J. Ethnopharmacol..

[29] Khan K.U., Minhas M.U., Sohail M., Badshah S.F., Abdullah O., Khan S., Munir A., Suhail M. (2021). Synthesis of PEG-4000-co-poly (AMPS) nanogels by cross-linking polymerization as highly responsive networks for enhancement in meloxicam solubility. Drug Dev. Ind. Pharm..

[30] Tokgöz S., Kara A., Peksoz A. (2020). Synthesis and characterization of poly (EGDMA-co-VPCA)/SWCNT composite films by surface polymerization method. Mater. Sci. Semicond. Process..

[31] Liu X., Zeng W., Zhao J., Qiu X., Xiong H., Liang Y., Ye X., Lei Z., Chen D. (2021). Preparation and anti-leakage properties of hydroxyethyl cellulose-g-poly (butyl acrylate-co-vinyl acetate) emulsion. Carbohydr. Polym..

[32] Zhang W., Hu Y., Zhao J., Zhang Y., Guo D., Gao C., Duan J., Li P. (2020). Immunoregulation and antioxidant activities of a novel acidic polysaccharide from Radix Paeoniae Alba. Glycoconj. J..

[33] Enawgaw H., Tesfaye T., Yilma K.T., Limeneh D.Y. (2021). Synthesis of a cellulose-co-amps hydrogel for personal hygiene applications using cellulose extracted from corncobs. Gels.

[34] Ayouch I., Kassem I., Kassab Z., Barrak I., Barhoun A., Jacquemin J., Draoui K., El Achaby M. (2021). Crosslinked carboxymethyl cellulose-hydroxyethyl cellulose hydrogel films for adsorption of cadmium and methylene blue from aqueous solutions. Surf. Interfaces.

[35] Tronci G., Ajiro H., Russell S.J., Wood D.J., Akashi M. (2014). Tunable drug-loading capability of chitosan hydrogels with varied network architectures. Acta Biomater..

[36] Owusu-Ware S.K., Boateng J., Jordan D., Portefaix S., Tasseto R., Ramano C.D., Antonijević M.D. (2016). Molecular mobility of hydroxyethyl cellulose (HEC) films characterised by thermally stimulated currents (TSC) spectroscopy. Int. J. Pharm..

[37] Ashames A., Ullah K., Al-Tabakha M., Khan S.A., Hassan N., Mannan A., Ikram M., Buabeid M., Murtaza G. (2022). Development, characterization and In-vitro evaluation of guar gum based new polymeric matrices for controlled delivery using metformin HCl as model drug. PLoS ONE.

[38] Farid-ul-Haq M., Amin M., Hussain M.A., Sher M., Khan T.A., Kausar F., Amin H.M. (2020). Comparative isoconversional thermal analysis of Artemisia vulgaris hydrogel and its acetates; a potential matrix for sustained drug delivery. Int. J. Polym. Anal. Charact..

[39] El Fawal G., Hong H., Song X., Wu J., Sun M., He C., Mo X., Jiang Y., Wang H. (2020). Fabrication of antimicrobial films based on hydroxyethylcellulose and ZnO for food packaging application. Food Packag. Shelf Life.

[40] Salama H.E., Aziz M.S.A. (2020). Novel biocompatible and antimicrobial supramolecular O-carboxymethyl chitosan biguanidine/zinc physical hydrogels. Int. J. Biol. Macromol..

[41] Salawi A., Khan A., Zaman M., Riaz T., Ihsan H., Butt M.H., Aman W., Khan R., Majeed I., Almoshari Y. (2022). Development of Statistically Optimized Chemically Cross-Linked Hydrogel for the Sustained-Release Delivery of Favipiravir. Polymers.

[42] Hosary R.E., El-Mancy S.M., El Deeb K.S., Eid H.H., Tantawy M.E.E., Shams M.M., Samir R., Assar N.H., Sleem A.A. (2020). Efficient wound healing composite hydrogel using Egyptian *Avena sativa* L. polysaccharide containing β-glucan. Int. J. Biol. Macromol..

[43] Zulkifli F.H., Hussain F.S.J., Harun W., Yusoff M.M. (2019). Highly porous of hydroxyethyl cellulose biocomposite scaffolds for tissue engineering. Int. J. Biol. Macromol..

[44] Khanum H., Ullah K., Murtaza G., Khan S.A. (2018). Fabrication and in vitro characterization of HPMC-g-poly (AMPS) hydrogels loaded with loxoprofen sodium. Int. J. Biol. Macromol..

[45] Stoyneva V., Momekova D., Kostova B., Petrov P. (2014). Stimuli sensitive super-macroporous cryogels based on photo-crosslinked 2-hydroxyethylcellulose and chitosan. Carbohydr. Polym..

[46] Noreen A., Zia K.M., Tabasum S., Khalid S., Shareef R. (2020). A review on grafting of hydroxyethylcellulose for versatile applications. Int. J. Biol. Macromol..

[47] Xing Z., Lu H., Hossain M. (2021). Renormalized Flory-Huggins lattice model of physicochemical kinetics and dynamic complexity in self-healing double-network hydrogel. J. Appl. Polym. Sci..

[48] Jalil A., Khan S., Naeem F., Haider M.S., Sarwar S., Riaz A., Ranjha N.M. (2017). The structural, morphological and thermal properties of grafted pH-sensitive interpenetrating highly porous polymeric composites of sodium alginate/acrylic acid copolymers for controlled delivery of diclofenac potassium. Des. Monomers Polym..

[49] Pandey S., Do J.Y., Kim J., Kang M. (2020). Fast and highly efficient removal of dye from aqueous solution using natural locust bean gum based hydrogels as adsorbent. Int. J. Biol. Macromol..

[50] Karoyo A.H., Wilson L.D. (2021). A review on the design and hydration properties of natural polymer-based hydrogels. Materials.

[51] Ferrero C., Massuelle D., Doelker E. (2010). Towards elucidation of the drug release mechanism from compressed hydrophilic matrices made of cellulose ethers. II. Evaluation of a possible swelling-controlled drug release mechanism using dimensionless analysis. J. Control. Release.

[52] Malik N.S., Ahmad M., Minhas M.U., Tulain R., Barkat K., Khalid I., Khalid Q. (2020). Chitosan/xanthan gum based hydrogels as potential carrier for an antiviral drug: Fabrication, characterization, and safety evaluation. Front. Chem..

[53] Hanna D.H., Lotfy V.F., Basta A.H., Saad G.R. (2020). Comparative evaluation for controlling release of niacin from protein-and cellulose-chitosan based hydrogels. Int. J. Biol. Macromol..

[54] Shakya S., Danshiitsoodol N., Sugimoto S., Noda M., Sugiyama M. (2021). Anti-oxidant and anti-inflammatory substance generated newly in Paeoniae Radix Alba extract fermented with plant-derived Lactobacillus brevis 174A. Antioxidants.

[55] Alvarez G.S., Hélary C., Mebert A.M., Wang X., Coradin T., Desimone M.F. (2014). Antibiotic-loaded silica nanoparticle–collagen composite hydrogels with prolonged antimicrobial activity for wound infection prevention. J. Mater. Chem. B.

[56] Guan Y., Yu C., Zang Z., Wan X., Naeem A., Zhang R., Zhu W. (2023). Chitosan/xanthan gum-based (Hydroxypropyl methylcellulose-co-2-Acrylamido-2-methylpropane sulfonic acid) interpenetrating hydrogels for controlled release of amorphous solid dispersion of bioactive constituents of Pueraria lobatae. Int. J. Biol. Macromol..

[57] Luo N., Li Z., Qian D., Qian Y., Guo J., Duan J.-A., Zhu M. (2014). Simultaneous determination of bioactive components of Radix Angelicae Sinensis–Radix Paeoniae Alba herb couple in rat plasma and tissues by UPLC–MS/MS and its application to pharmacokinetics and tissue distribution. J. Chromatogr. B.

[58] Yu C., Chen X., Zhu W., Li L., Peng M., Zhong Y., Naeem A., Zang Z., Guan Y. (2022). Synthesis of Gallic Acid-Loaded Chitosan-Grafted-2-Acrylamido-2-Methylpropane Sulfonic Acid Hydrogels for Oral Controlled Drug Delivery: In Vitro Biodegradation, Antioxidant, and Antibacterial Effects. Gels.

[59] Naeem A., Yu C., Zhu W., Zang Z., Guan Y. (2023). Study of Hydroxypropyl β-Cyclodextrin and Puerarin Inclusion Complexes Encapsulated in Sodium Alginate-Grafted 2-Acrylamido-2-Methyl-1-Propane Sulfonic Acid Hydrogels for Oral Controlled Drug Delivery. Gels.

[60] Naeem A., Yu C., Zhu W., Chen X., Wu X., Chen L., Zang Z., Guan Y. (2022). Gallic Acid-Loaded Sodium Alginate-Based (Polyvinyl Alcohol-Co-Acrylic Acid) Hydrogel Membranes for Cutaneous Wound Healing: Synthesis and Characterization. Molecules.

[61] Hanna D.H., Saad G.R. (2019). Encapsulation of ciprofloxacin within modified xanthan gum-chitosan based hydrogel for drug delivery. Bioorg. Chem..

[62] Iqbal F.M., Iqbal S., Nasir B., Hassan W., Ahmed H., Iftikhar S.Y. (2022). Formulation of captopril-loaded hydrogel by microwave-assisted free radical polymerization and its evaluation. Polym. Bull..

[63] Kesharwani P., Fatima M., Singh V., Sheikh A., Almalki W.H., Gajbhiye V., Sahebkar A. (2022). Itraconazole and Difluorinated-Curcumin Containing Chitosan Nanoparticle Loaded Hydrogel for Amelioration of Onychomycosis. Biomimetics.

[64] Pandey M., Choudhury H., D/O Segar Singh S.K., Chetty Annan N., Bhattamisra S.K., Gorain B., Mohd Amin M.C.I. (2021). Budesonide-loaded pectin/polyacrylamide hydrogel for sustained delivery: Fabrication, characterization and in vitro release kinetics. Molecules.

[65] Ilgin P., Ozay H., Ozay O. (2019). A new dual stimuli responsive hydrogel: Modeling approaches for the prediction of drug loading and release profile. Eur. Polym. J..

[66] Pettinelli N., Rodriguez-Llamazares S., Abella V., Barral L., Bouza R., Farrag Y., Lago F. (2019). Entrapment of chitosan, pectin or κ-carrageenan within methacrylate based hydrogels: Effect on swelling and mechanical properties. Mater. Sci. Eng. C.

[67] Naeem A., Yu C., Zang Z., Zhu W., Deng X., Guan Y. (2023). Synthesis and Evaluation of Rutin–Hydroxypropyl β-Cyclodextrin Inclusion Complexes Embedded in Xanthan Gum-Based (HPMC-g-AMPS) Hydrogels for Oral Controlled Drug Delivery. Antioxidants.

[68] Hegab R.A., Pardue S., Shen X., Kevil C., Peppas N.A., Caldorera-Moore M.E. (2020). Effect of network mesh size and swelling to the drug delivery from pH responsive hydrogels. J. Appl. Polym. Sci..

[69] Zhao J., Sun C., Li H., Dong X., Zhang X. (2020). Studies on the physicochemical properties, gelling behavior and drug release performance of agar/κ-carrageenan mixed hydrogels. Int. J. Biol. Macromol..

[70] Zang Z., Zhao S., Yang M., Yu C., Ouyang H., Chen L., Zhu W., Liao Z.-G., Naeem A., Guan Y. (2022). Blood chemical components analysis of honeysuckle and formulation of xanthan gum/starch-based (PVA-co-AA) hydrogels for controlled release. Arab. J. Chem..

[71] Peppas N.A., Barr-Howell B.D. (2019). Characterization of the cross-linked structure of hydrogels. Hydrogels in Medicine and Pharmacy.

[72] Qu J., Zhao X., Liang Y., Xu Y., Ma P.X., Guo B. (2019). Degradable conductive injectable hydrogels as novel antibacterial, anti-oxidant wound dressings for wound healing. Chem. Eng. J..

[73] Esposito L., Barbosa A.I., Moniz T., Costa Lima S., Costa P., Celia C., Reis S. (2020). Design and characterization of sodium alginate and poly (vinyl) alcohol hydrogels for enhanced skin delivery of quercetin. Pharmaceutics.

[74] Ćorković I., Pichler A., Buljeta I., Šimunović J., Kopjar M. (2021). Carboxymethylcellulose hydrogels: Effect of its different amount on preservation of tart cherry anthocyanins and polyphenols. Curr. Plant Biol..

[75] Khattak S., Qin X.-T., Huang L.-H., Xie Y.-Y., Jia S.-R., Zhong C. (2021). Preparation and characterization of antibacterial bacterial cellulose/chitosan hydrogels impregnated with silver sulfadiazine. Int. J. Biol. Macromol..

